# Strengthening service integration for effective linkage of HIV-positive mothers to antiretroviral treatment: a cross-sectional study in two military health facilities in Kaduna, Nigeria, 2014

**DOI:** 10.11604/pamj.supp.2019.32.1.13315

**Published:** 2019-01-25

**Authors:** Fatima Ladidi Cheshi, Patrick Mboya Nguku, Ndadilnasiya Endie Waziri, Kabir Sabitu, Ojor Robinson Ayemoba, Tahir Oshe Umar, Peter Nsubuga

**Affiliations:** 1 Nigeria Field Epidemiology and Laboratory Training Program (NFELTP), Abuja, Nigeria; 2 Department of Community Medicine, Ahmadu Bello University, Zaria, Nigeria; 3 Ministry of Defence Health Implementation Programme, Abuja, Nigeria; 4 Global Public Health Solutions, Atlanta GA, USA

**Keywords:** Service integration, PMTCT, referral, linkage

## Abstract

**Introduction:**

strong PMTCT-ART service linkages ensure continuity of care for healthier mothers and children born HIV free. Program data showed weak PMTCT- ART linkages in military health facilities. We conducted a study to assess the PMTCT-adult ART service linkage in two Nigerian military health facilities in Kaduna State.

**Methods:**

we conducted a cross-sectional study using mixed methods (interviews and FGDs) in 44 Nigeria Army Reference Hospital (NARH) and 1 Division Hospital, Kaduna. We studied 372 HIV-positive mothers after a delivery of their babies, referred for ART services from January 2009 to December 2013. We conducted FGDs among ANC, PMTCT and ART clinics staff. We analysed data using descriptive and inferential methods. A p-value of < 0.05 was considered significant with 95% confidence intervals (CI) for estimates.

**Results:**

of the 372 respondents studied, 320 (86%) accessed PMTCT services from the 44 NARH. Most respondents (206,55.4%) respondents aged < 25 years. One in six (16.7%) respondents had no record of referral. Delivering baby in a separate facility from where PMTCT services were accessed, increased the likelihood of not accessing ART services (odd ratio [OR]: 6.7, 95% CI= 3.3 -13.6). The qualitative study identified poor service integration between PMTCT and ANC clinics.

**Conclusion:**

the key factors hindering PMTCT-ART linkage in military health facilities included poor service integration, clients delivering of a baby in a facility separate from where PMTCT services were accessed. The Ministry of Defence HIV programme should strengthen ANC-PMTCT-ART service integration through a centrally coordinated client information management system.

## Introduction

HIV infection is one of the leading causes of death among women of child-bearing age [1]. In 2014, sub-Saharan Africa accounted for nearly 85% of the global burden and remained the region with the largest proportion of pregnant women living with HIV [2]. The Prevention of Mother to Child Transmission (PMTCT) program provides antiretroviral prophylaxis for HIV-positive pregnant women during pregnancy, labor, delivery and postpartum periods [3]. Despite the scale up of PMTCT interventions, linkage of the mother and baby to comprehensive HIV/AIDS care after delivery has remained a challenge especially in developing countries in this region [4, 5]. Most eligible HIV-positive pregnant women, face significant delays starting antiretroviral therapy (ART) when referred [5-7]. Strong linkages between PMTCT and the adult ART clinic would ensure continuity of care resulting in healthier mothers and children born free of HIV. Service integration is, therefore, important in countries with generalized HIV epidemics (i.e., with HIV prevalence rate of > 1% in the general population) while minimizing social and structural impediments to service delivery [8].

Globally, the PMTCT strategy has evolved into the elimination of mother to child transmission (eMTCT) of HIV, with the World Health Organization (WHO) recommended: “Option B+” in which all HIV-infected pregnant women receive triple ART for life irrespective of the CD4 count levels . This differs from the Option B which places a women on ARVs depending on CD4 count levels. The Option B+ is tailored to address challenges with CD4 testing and assessment of ART eligibility at the primary level of health care; while providing a simpler one-regime approach (i.e., a single tablet fixed-dose combination (FDC) regimen) for all pregnant women. This option facilitates the full linkage of ART and PMTCT programs [9, 10].

In 2014, Nigeria had a median HIV prevalence of 4.1% amongst pregnant women attending antenatal clinics from ANC sentinel survey [11]. Nationally, the current PMTCT guidelines and clinical protocols use the “Option B” approach. Option B provides ART to HIV-positive pregnant and lactating women. The initiation of a woman on ART depends on certain biomarkers, such as CD4 count. However, some women stop the medication, only to restart later, when CD4 count drops below 350 cells/ml. Nigeria is yet to adopt the Option B+, as it is estimated to be more expensive than Option B. As at the time of the study, the HIV program of the Nigerian Military was using the “Option B” strategy, with 46 health facilities comprising 23 comprehensive and 13 satellite health facilities offering PMTCT services. Evidence from programme data in military health facilities showed weak PMTCT- adult ART referral at these facilities without a clear understanding of the reasons responsible for the situation.

## Methods

**Study design:** we conducted a cross-sectional study using mixed methods (interviews and FGDs), comprising a cross-sectional study (quantitative component) and focus group discussions (qualitative component) in two selected military health facilities.

**Study area:** the Nigeria Ministry of Defence, HIV/AIDS programme provides PMTCT services at 38 military health facilities across Nigeria, of these facilities 23 are comprehensive sites providing on-site ART services. The remaining 15 facilities are satellite sites, of which 13 facilities provided only PMTCT services and two (2) facilities offering only HCTservices , without on-site ART services, hence refer clients who need to begin ART to one of the 23 comprehensive facilities. However, eight (8) out of the 23 (twenty three) comprehensive sites do not have satellite sites attached to them. We conducted the study in 44 Nigeria Army Reference Hospital Kaduna, a “comprehensive facility” that provides HIV counseling and testing (HCT), PMTCT and ART services and 1 Division Hospital Kaduna a “satellite facility” that provides only HCT and PMTCT”. Both facilities are located in the Northwest zone of the country. Military and civilian clients from across Nigeria access these facilities.

**Figure 1:**
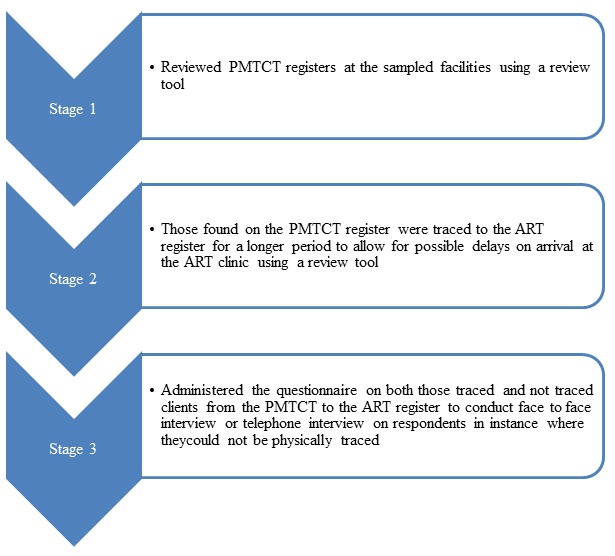
chart showing the stages in data collection process during the study assessing the referral linkage

**Study population:** HIV-positive pregnant women who accessed PMTCT services and were referred for ART services, following the delivery of their babies, from January 2009 to December 2013 and health care workers at the sampled clinics involved in the provision of HCT, PMTCT and ART services constituted the study population. The clients’ referral flow from ANC and PMTCT clinics in both the satellite and comprehensive site to the ART clinic at the comprehensive site was as shown in Figure 1. Clients either were self-referred or were referred from the TB clinic at the comprehensive site, private health facilities and community to the PMTCT clinic. At postnatal visits, the HIV-positive mothers were referred to the ART clinic for their health, ensuring a continuum of care.

### Eligibilty criteria

Inclusion criteria: we included all HIV positive mothers who attended ANC, received PMTCT services and accessed ART services following referral to continue ART after the delivery of their babies, as indicated on the ANC, PMTCT and ART clinic registers, and also all HIV positive mothers who had been referred to the two health facilities at least six months prior to the study who met the same criteria above, to allow enough time to ascertain defaulters.

**Exclusion criteria:** we excluded mothers who were unreachable by phone during data collection and those with missing CD4 count records on the PMTCT registers and those who did not give informed consent.

**Sample size:** our minimum sample size was 372 using the Fisher *et al*., 1998 formula [12]:

$$\text{n}=\frac{\text{z}\alpha^{2}\text{pq}}{{\text{d}}^{2}}$$

(n = minimum sample size); z = 1.96, standard normal deviation at 95% confidence interval; a = 0.05 (2 sided), probability of type 1 error; p (women who defaulted on referral in a health facility from a previous study in Tanzania) = 68.0%; q = 1-p, is the proportion of women who were linked to ART services in a referral health facility in Tanzania from a previous study = 32.0%; [13] and increased the sample by 10% to cater for non-response rate.

**Sampling technique:** we randomly selected one comprehensive facility from a sample frame of all the comprehensive facilities within Nigerian Military HIV programme. We then randomly selected one satellite facility from a list of satellite facilities attached to the earlier sampled comprehensive facility by balloting. Respondents were selected from the PMTCT registers using systematic random sampling. The client load accessing PMTCT/ART services at these facilities was used to determine the proportion of respondents from each facility (one-third from the satellite and two-third from the comprehensive site). The first respondent was selected using simple random method via a table of random numbers. Every 5th client was selected until the desired sample size was attained. We selected staff working at the PMTCT and ART clinic to participate in focus group discussions.

**Data collection and storage:** the process of data collection was carried out in three stages as shown in Figure 1. We also conducted three focus group discussions in a conducive environment, among the doctors, nurses and health record officers at the ANC, PMTCT and ART clinics. Each focus group discussion had 6-12 consenting respondents. Discussion notes were captured by a note taker, recorded using a tape recorder and subsequently transcribed into notes for analysis. We collected data using Microsoft Excel and exported to Epi InfoTM version 7.0 (US Centers for Disease Control and Prevention) for analysis. Each respondent had a unique identification number on the spreadsheet. The respondents’ records and all identifiers were stored in a separate database with a password-protected laptop.

**Data analysis:** we conducted univariate, bivariate, multivariate (unconditional logistic regression) analyses. The dependent variables assessed were ART referral linkage and ART uptake while the independent variables included age, marital status, educational status, employment status, clinic type attended for PMTCT, referral status, form of referral, place of delivery of baby and referring personnel. A p-value of < 0.05 was considered significant and used to compare between HIV positive mothers who were linked and those not linked to ART services from the two selected facilities, with 95% confidence intervals (CI) for estimates. We compared two groups based on ART linkage using odds ratios. Qualitative data from focused group discussions were analyzed using content analysis.

**Ethical clearance:** ethical clearance was obtained from the Ministry of Defence Health Research Ethics Committee and the ethical committee of Kaduna State Ministry of Health. We obtained written and/or verbal consent from all respondents while ensuring confidentiality.

## Results

Out of the 372 respondents that were studied, 52 (14%) of those that accessed PMTCT services were from the satellite site and 320 (86%) who accessed PMTCT came from the comprehensive site. A total of 62 (16.7%) of the respondents had no record of being referred. A total of 316 women were linked to ART while the remaining 56 were not linked. There were 206 (55.4%) respondents aged < 25 years. The mean age of respondents not linked to ART was 30.9 years, standard deviation (SD) ±4.9 years while for those linked to ART was 31.4 years ± SD 4.6 years. The majority of those not linked to ART (42/56, 74.7%) and those linked to ART, (203/245, 64.3%) were married or cohabiting (Table 1).

**Table 1:** socio-demographic characteristics of respondents in PMTCT –ART referral linkage study in two military health facilities, Kaduna, Nigeria, 2014

Variables	Not linked to ART services and uptake (n=56) (%)	Linked to ART services and uptake (n=316) (%)
**Age (years)**		
Mean (±SD)	30.9 (±4.9)	31.4 (±4.6)
**Marital status**		
Married/cohabiting	42 (74.7)	203(64.3)
Single/divorced/widowed	14 (25.3)	113 (35.7)
**Educational status**		
None/primary	6 (10.7)	28 (8.9)
Secondary/tertiary	50 (89.3)	288 (91.1)
**Employment status**		
Unemployed	21 (37.5)	131 (41.5)
Employed	35 (62.5)	185 (58.5)
**PMTCT clinic**		
Comprehensive site	50 (89.2)	215 (67.9)
Satellite site	6 (10.8)	101 (32.1)

### Bivariate analysis of predictors for ART default by mothers

Delivering of a baby outside ANC and PMTCT facilities increased the likelihood of not accessing ART services (OR: 6.7, 95% CI = 3.3 -13.6). A client who fulfilled the CD4 eligibility criteria of ≥ 350 cells/ml was found to be more likely not to be referred based on guidelines and hence not to assess ART [OR: 0.02, 95% Confidence Interval (CI) = 0.003 -0.138] (Table 2). Of the 147 eligible women traced to ART clinic, one in six (16.7%) respondents were found to be without record of formal referral.

**Table 2:** bivariate analysis showing factors associated with referral linkage in two Nigeria military health facilities, Kaduna, January 2009-December 2013

Variable	Not Linked to ART services Frequency (%)	Linked to ART services Frequency (%)	Odds Ratio (95% CI)
**Place of delivery of baby**			
Outside PMTCT facility	18(32.1)	21(6.6)	6.7(3.3-13.6)
In PMTCT facility	38(67.9)	295(93.4)	
**CD4 eligibility**			
≥ 350 cells/ml	1(1.8)	155(49.1)	0.02(0.003-0.14)
< 350 cells/ml	55(98.2)	161(50.9)	
**Referring Personnel**			
Nurse/Lab staff/Counselor	44(78.6)	203(64.2)	2.0(1.0-4.0)
Doctor	12(21.4)	113(35.8)	
**Knowledge of reason for referral**			0.5(0.2-1.6)
Not knowing why referred	4(7.1)	39(12.3)	
Knowing why referred	52(92.9)	277(87.7)	
**Form of referral**			
Written	25(44.6)	119(37.7)	1.3(0.8-2.4)
Oral	31(55.4)	197(62.3)	
**Support group membership**			
Non-member	34(60.7)	205(64.9)	0.8(0.5-1.5)
Member	22(39.3)	111(35.1)	

**Figure 2:**
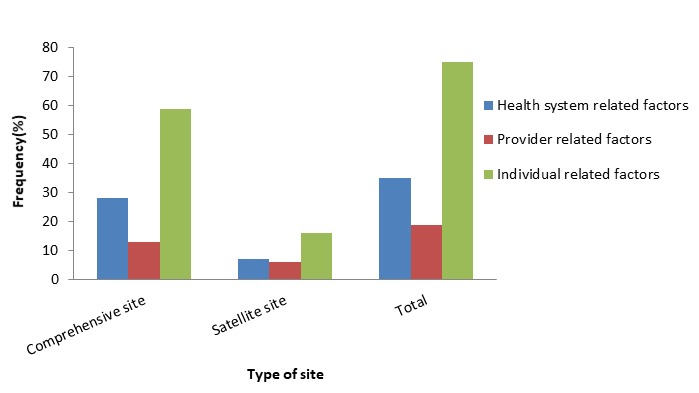
factors associated with PMTCT- ART referral linkage by type of site

The breakdown by type of site of the factors associated with PMTCT-ART referral linkage is shown in Figure 2. Individual related factors such as: marital status, religion, educational level, employment status, were the most likely to be associated with not being linked to ART services in the sampled facilities. Health system related factors such as: referral status of clients and form of referral (either oral or written form) ranked second. Provider related factors such as referring health personnel, stigmatization by a health worker, long waiting time at the facility and unfriendly health workers at the health facility were least likely to be associated with not being linked to ART services. Marital status, place of delivery of baby, knowledge of the reason for referral, referring personnel and CD4 cell count eligibility were not associated with PMTCT-ART linkage in the multivariable logistic regression model (Table 3).

**Table 3:** multivariate analysis of factors affecting PMTCT-ART referral linkage, in two Nigeria military health facilities, Kaduna, January 2009-December 2013

Factors affecting PMTCT- ART referral linkage	Adjusted odds ratio(CI)
Delivery of baby outside PMTCT facility	0.79(0.18-3.42)
Married/Co-habiting	0.92(0.25-3.38)
Not being referred by a doctor	1.22(0.32-4.60)
Not knowing the reason for referral	2.72(0.36-20.34)

### Qualitative study

From the FGDs, place of delivery of the baby, poor ANC-PMTCT service integration, self-stigmatization, non-disclosure to partner and security challenges were likely factors influencing referral linkages. The following five themes were identified from the FGDs as factors affecting PMTCT-ART linkages.

**Theme 1: failure of clients to return for CD4 cell count result:** some clients were not returning for their CD4 count result at the comprehensive facility that had served as a referral facility for some public and private hospitals in the state and environs. Quote: “Some clients do not return for their CD4 count results. Hence, it becomes difficult to categorize the clients into those that need ART and those that need adult care and support only”.

**Theme 2: self-stigmatization is still a factor that affects referral between:** PMTCT and ART clinics. Quote: “Patients hide and refuse to go to the facility because of self-stigma. Some clients not booked at the labor ward refuse to reveal their status, so go unidentified”.

**Theme 3: non-disclosure to partner:** women who came for antenatal care were quite comfortable to access other services provided including PMTCT care while they were pregnant. However, after delivery of their babies, accessing ART continued care at the ART clini became a challenge for those that had not disclosed their status to their partners. Quote: “Non-disclosure is a very big challenge that hampers clients follow up”.

**Theme 4: low hospital delivery rate:** there was difficulty in ensuring that all women who attended the facilities for ANC and PMTCT services to return to deliver their babies at the same facilities. Quote: “Not all the women attending ANC in this facility return for delivery and referral in this facility where they accessed ANC and PMTCT services”.

**Theme 5: poor service integration between PMTCT and the ANC clinics:** there were reports of duplication of records with every HIV-positive pregnant client having two client folders, one for the PMTCT and another for the ANC service delivery points. The service providers at these two points did not usually compare notes hence resulting in a poor continuum of care. Quote: “Each HIV-positive pregnant woman that accesses this facility has two folders, one in the ANC and one in the PMTCT clinic”.

## Discussion

The mixed methods study of HIV-positive pregnant mothers was conducted to determine the proportion of women accessing PMTCT who were referred to the adult ART clinic, the proportion of referred HIV positive mothers who reached and accessed ART services, identify the factors associated with PMTCT-ART service linkage in selected Nigerian military health facilities.

We found that linked and unlinked clients to ART services were similar in age group. A larger proportion of the respondents were < 25 years old, which is reflective of early initiation of childbearing in northern Nigeria. The clients referred from the satellite clinic to the ART clinic at the comprehensive facility may have defaulted on referral while client referred within the comprehensive facility, were more likely to access the ART service due to the proximity. This finding was consistent with findings in other studies where clients were more likely to access ART service which within the same location as the PMTCT clinic, underscoring the role of service integration in referral linkage of clients [13, 14].

Despite the high referral rate from this study, it still falls short of the 100% referral rate target recommended by the national guidelines [3]. Our study showed evidence that not all HIV positive mothers were referred to ART clinic, this could be due to incomplete data on client referral status or health care provider not referring a client. This result we found is consistent with a Zambian study that revealed a capacity building of human resource resulting in an increased referral rate by over 40% [15]. Also, a woman delivering her baby outside the PMTCT facility made her less likely to access ART than those who had their babies in the same facility as the PMTCT clinic. The result we found is consistent with a Tanzanian study and supported by the findings of our FGD, where women attended ANC , PMTCT and returned for delivery and referral at the same facility [13, 16]. We also found a mode of referral and support group membership not associated with effective referral linkage, differing from findings of a Tanzanian study where support group membership made a client twice more likely to assess ART services than non-members [13].

Focus group discussants pointed out service integration in the referral health facility as a major factor influencing referral linkage of HIV-positive mothers from the PMTCT to the ART clinic. The majority of women who were not referred were from the comprehensive facility and lost the opportunity to access ART services. Hence, the satellite facility was more efficient in the referral of clients. The majority of the women who were linked to the comprehensive ART clinic were referred from the PMTCT clinic of the comprehensive facility. This finding was consistent with findings in other studies where service integration of services was found to result to higher rates of ART enrolment and ART coverage [17]. We found knowledge and understanding of why a client was referred had no association with the weak linkage; this differs from a Zimbabwean study, where understanding the referral process made a client three times more likely to access ART services than those who did not understand the process [18].

We recognize four limitations to generalizing the results of our study. Being facility-based made representativeness a challenge. Incomplete PMTCT and ART data may have resulted in information bias to the study. Absence or incomplete home address of most clients made it difficult to trace all the defaulters to their homes. Hence, we conducted telephone interviews for those who we were unable to trace home. The cross-sectional study design made establishing causality difficult.

## Conclusion

Health system, provider-related and individual factors were associated with poor PMTCT and ART referral linkage. Poor service integration with disconnection among ANC, PMTCT and ART service providers, poor client utilization of the same facility for ANC, PMTCT and delivery of their babies were key factors identified as affecting PMTCT and ART referral linkage. We recommend that the Ministry of Defence HIV control programme with support from the Federal Ministry of Health (FMOH) should strengthening ANC-PMTCT-ART service integration through a centrally coordinated client information management system in these Nigeria military facilities, establishing an active tracking system for defaulters in these facilities and introducing incentives for HIV pregnant women to create demand for delivery services in the same facility as PMTCT clinic.

### What is known about this topic

- The reasons for weak PMTCT- ART referral linkages can be multifactorial;
- Active follow up of defaulters improves PMTCT- ART referral linkages;
- HIV- related stigma is a significant factor associated with poor PMTCT- ART referral linkage.

### What this study adds

- Service integration is a strong backbone for good effective PMTCT- ART referral linkage;
- Close proximity alone does not necessarily ensure effective PMTCT- ART referral linkage of clients;
- Individual factors influencing PMTCT- ART referral linkage seems to be more influential than systemic and provider-related factors.

## Competing interests

The authors declare no competing interests.
